# Case Report: Clinical implications of *HLA*-B*15:02 screening in pediatric patients: a case series from a Thai Binh Children's Hospital, Vietnam

**DOI:** 10.3389/fped.2026.1825798

**Published:** 2026-05-15

**Authors:** Thi Huong Pham, Trong Kiem Tran, Manh Dung Do, Thi Hoa Luu, Thi Ngoc Hoa Doan, Thi Ha Le, Thi Phuong Mai Truong

**Affiliations:** 1 Thai Binh Pediatric Hospital, Thai Binh, Vietnam; 2 Thai Binh University of Medicine and Pharmacy, Thai Binh, Vietnam

**Keywords:** carbamazepine, *HLA*-B*15:02, pediatric neurology, personalised medicine, pharmacogenetics, Stevens-Johnson syndrome, Vietnam

## Abstract

**Background:**

Carbamazepine (CBZ) is a widely used antiseizure medication, but its use is associated with severe cutaneous adverse reactions (SCARs), including Stevens-Johnson syndrome (SJS) and toxic epidermal necrolysis (TEN). In Southeast Asian populations, including Vietnam, *HLA*-B*15:02 is a well-established genetic marker associated with CBZ-induced SJS/TEN. Although pre-treatment screening is recommended in high-risk populations, implementation in provincial hospitals remains limited. This study evaluated the clinical utility of *HLA*-B*15:02 screening and the observed frequency of the allele in a preliminary cohort of pediatric patients at Thai Binh Children's Hospital.

**Materials and methods:**

We conducted a descriptive case series of 14 consecutive pediatric patients aged 4 to 15 years who were considered for CBZ therapy at Thai Binh Children's Hospital. *HLA*-B*15:02 screening was performed using the GenePx 8 diagnostic system. Demographic characteristics, screening results, and post-screening treatment decisions were recorded and analyzed. Written informed consent for genetic testing and the use of de-identified clinical data for publication was obtained from parents or legal guardians.

**Results:**

Among the 14 patients screened (8 females, 57.1%; 6 males, 42.9%), 3 patients (21.4%) tested positive for *HLA*-B*15:02. These positive cases included one male and two females born between 2013 and 2019. In all genotype-positive cases, clinicians changed the planned treatment and prescribed alternative antiseizure medications instead of CBZ. No cases of SJS/TEN or other severe cutaneous adverse reactions were observed during the initial follow-up period.

**Conclusion:**

In this small preliminary case series, *HLA*-B*15:02 screening was feasible in a provincial hospital and may assist safer prescribing of CBZ in pediatric practice. The observed frequency in this cohort should not be interpreted as a population prevalence estimate. Larger multicenter studies are needed to confirm these findings and to assess broader clinical and economic implications.

## 1 Introduction

Carbamazepine (CBZ) remains an important treatment option for pediatric epilepsy and some neuropathic pain conditions because of its clinical efficacy and relatively low cost. However, its use is limited by the risk of severe cutaneous adverse reactions (SCARs), particularly Stevens-Johnson syndrome (SJS) and toxic epidermal necrolysis (TEN) (1). These reactions may cause extensive epidermal detachment, multisystem complications, and substantial morbidity and mortality (2). In Southeast Asian populations, *HLA*-B*15:02 has been identified as a strong genetic marker associated with CBZ-induced SJS/TEN (3). Accordingly, international pharmacogenetic guidelines recommend pre-treatment screening in populations with a high allele frequency before CBZ is initiated (4).

In the Vietnamese population, the frequency of *HLA*-B*15:02 has been reported to be approximately 10% to 15% among the Kinh ethnic majority (5). Despite the established clinical relevance of this association, access to pharmacogenetic testing remains concentrated in tertiary centers, while implementation in provincial hospitals may be constrained by limited infrastructure, costs, and workflow challenges (6). For pediatric patients in such settings, the lack of timely screening may complicate treatment selection when CBZ is being considered (7).

The association between *HLA*-B*15:02 and CBZ-induced SJS/TEN is already well established. Therefore, the main contribution of this study is not to demonstrate a novel genetic association, but to describe the early real-world implementation of *HLA*-B*15:02 screening in a provincial pediatric hospital in Vietnam. We report a small descriptive case series focusing on the observed frequency of *HLA*-B*15:02 in this cohort, the feasibility of testing in routine care, and the immediate influence of test results on prescribing decisions.

## 2 Materials and methods

### 2.1 Study design and setting

A descriptive case series was conducted at Thai Binh Children's Hospital, a provincial pediatric hospital in northern Vietnam. The study focused on the initial implementation of *HLA*-B*15:02 screening in routine pediatric practice before consideration of CBZ therapy.

### 2.2 Study subjects

The study included 14 pediatric patients who met the following criteria: (i) aged 0 to 16 years; (ii) considered for CBZ therapy for conditions such as epilepsy or neuropathic pain; and (iii) underwent *HLA*-B*15:02 screening as part of the hospital's medication-safety protocol in accordance with CPIC guidance (4). Patients whose parents or legal guardians did not consent to genetic testing or to the use of de-identified data for publication were excluded.

### 2.3 Sample size and sampling method

A consecutive sampling approach was used. The study included all pediatric patients (*n* = 14) who underwent *HLA*-B*15:02 testing during the initial implementation phase of the screening program at Thai Binh Children's Hospital. Because of the descriptive case-series design and small sample size, the study was intended to provide an initial feasibility-oriented clinical description rather than a population-level prevalence estimate.

### 2.4 Data acquisition and variable selection

Data were collected from the hospital's electronic medical records and laboratory information system using a standardised case report form. Extracted variables included age, sex, clinical indication for considering CBZ therapy, *HLA*-B*15:02 test result, and post-screening treatment decisions. The primary outcome was the presence or absence of *HLA*-B*15:02. Secondary outcomes were treatment changes following receipt of the genetic test result.

### 2.5 Laboratory infrastructure and genotyping protocol

All genomic analyses were conducted at the Molecular Biology Laboratory of Thai Binh Children's Hospital. Peripheral venous blood samples (2 mL) were collected in K2-EDTA tubes. Genotyping for *HLA*-B*15:02 was performed using the GenePx 8 system (Gene Solutions, Vietnam). According to the manufacturer's documentation, the platform integrates DNA extraction, amplification, and fluorescence-based detection in a closed workflow, with an expected turnaround time of approximately 90 to 120 min (8).

### 2.6 Statistical analysis

Data were analysed using IBM SPSS Statistics version 26.0 and Microsoft Excel 365. Categorical variables were summarised as frequencies and percentages. Given the small sample size, the findings were interpreted descriptively. The observed frequency of *HLA*-B*15:02 in this cohort was reported with a 95% confidence interval calculated using the Wilson score method.

### 2.7 Ethical consideration

The study protocol was reviewed and approved by the Institutional Review Board of Thai Binh Children's Hospital. All procedures were conducted in accordance with the Declaration of Helsinki. Written informed consent for genetic testing and for the use of anonymized clinical data in publication was obtained from the parents or legal guardians of all participating children. Patient confidentiality was protected by de-identifying all personal information.

## 3 Results

### 3.1 Demographic characteristics of the study cohort

A total of 14 pediatric patients were included in this case series. The cohort comprised 8 females (57.1%) and 6 males (42.9%). Birth years ranged from 2011 to 2022. These data describe the characteristics of the screened cohort only and were not intended to represent the broader pediatric population of Thai Binh province. Baseline demographic and screening characteristics of the cohort are presented in Table 1.

**Table 1:** Baseline demographics and genetic screening profiles (*n* = 14).

Characteristic	Frequency	Percentage (%)
Gender		
Male	6	42.9
Female	8	57.1
*HLA*-B*15:02 Status		
Positive	3	21.4
Negative	11	78.6
Age Group (Birth Year)		
Early Childhood (2019-2022)	5	35.7
Middle Childhood (2015-2018)	5	35.7
Late Childhood/Adolescence (2011-2014)	4	28.6

### 3.2 *HLA*-B*15:02 screening outcomes

The GenePx 8 system provided interpretable genotyping results for all 14 samples, and no repeat testing was required. The observed frequency of *HLA*-B*15:02 in this cohort was 21.4% (3/14; 95% CI: 7.5%–47.6%).

Among the three positive cases, two were female, and one was male. The positive results were observed in patients born in 2013, 2016, and 2019, indicating that the allele was present across multiple pediatric age groups in this small cohort.

### 3.3 Clinical management and therapeutic interventions

Screening results influenced treatment decisions in this cohort. For the 11 patients (78.6%) who tested negative, CBZ therapy was initiated as planned under standard clinical monitoring. No cases of SJS/TEN or other severe cutaneous adverse reactions were observed during the initial follow-up period.

In contrast, all three patients who tested positive for *HLA*-B*15:02 had their planned treatment changed. CBZ was not initiated, and alternative antiseizure medications were selected to reduce the risk of SCARs. The genotype-positive cases and the corresponding clinical actions are summarized in Table 2.

**Table 2:** Summary of *HLA*-B*15:02 positive cases and subsequent clinical actions.

Case ID	Gender	Birth year	Genotype	Clinical action	Alternative medication
1	Female	2013	Positive	CBZ Contraindicated	Valproate
2	Female	2016	Positive	CBZ Contraindicated	Levetiracetam
3	Male	2019	Positive	CBZ Contraindicated	Valproate/Topiramate

## 4 Discussion

### 4.1 Observed frequency of *HLA*-B*15:02 in the study cohort

In this case series, the observed frequency of *HLA*-B*15:02 was 21.4% (3/14) among pediatric patients for whom CBZ therapy was being considered at a provincial hospital in northern Vietnam. This proportion is higher than the approximately 10%–15% frequency previously reported in the Vietnamese Kinh population (5). However, because this was a small, single-center descriptive cohort, this finding should be interpreted cautiously and should not be considered a population prevalence estimate. The difference may reflect random variation, the clinical characteristics of the screened cohort, or broader HLA variation within the Kinh Vietnamese population (9). Findings from neighboring Southeast Asian populations also support the relevance of *HLA*-B*15:02 in CBZ safety assessment, particularly in Thai and Malaysian cohorts (10–12).

### 4.2 Clinical utility and immediate therapeutic impact

A key finding of this study was that all genotype-positive patients had their treatment plans changed after screening. In these cases, clinicians avoided CBZ and selected alternative antiseizure medications. Although our small descriptive study cannot directly quantify risk reduction, this genotype-informed approach may help reduce the likelihood of CBZ-related SJS/TEN. Conversely, genotype-negative patients were able to start CBZ under routine monitoring, which illustrates the practical prescribing value of targeted pharmacogenetic testing (13).

### 4.3 Feasibility of pharmacogenetics in provincial healthcare

This study also suggests that *HLA*-B*15:02 screening can be implemented at the provincial hospital level. Pharmacogenetic testing in routine care may be limited by infrastructure, logistics, and service-delivery barriers, particularly outside tertiary centers (6, 14). In our setting, the automated platform provided results within approximately 90 to 120 min, allowing treatment decisions to be made during the same clinical encounter or early during admission (8). These observations are consistent with broader efforts to integrate pharmacogenetic services into routine care, including in resource-limited settings (6, 14, 15).

### 4.4 Economic considerations and healthcare policy

Although this case series was not designed as a formal economic evaluation, the potential financial consequences of preventing a single case of SJS/TEN may be substantial because management often requires prolonged hospitalization and intensive supportive care (16). In resource-constrained settings, such considerations are relevant to policy discussions. Our findings therefore support further evaluation of whether *HLA*-B*15:02 screening should be incorporated into broader medication-safety policies for patients starting CBZ, especially in pediatric practice (17).

### 4.5 Limitations and future perspectives

This study has several important limitations. First, the sample size was very small (*n* = 14), which limits generalizability and precludes the use of this cohort to estimate population prevalence. Second, the study was conducted at a single center during the early implementation phase of testing. Third, follow-up was limited, and no comparator group was included. Finally, other pharmacogenetic markers that may be clinically relevant, such as *HLA*-A*31:01, were not evaluated (7). Larger multicenter studies are needed to confirm feasibility, assess broader outcomes, and determine the cost-effectiveness of routine screening in provincial hospitals.

## 5 Conclusion

In this small preliminary case series, *HLA*-B*15:02 screening was feasible in a provincial pediatric hospital and may assist safer prescribing before CBZ initiation. The observed frequency in this cohort should be interpreted cautiously and not as a population prevalence estimate. Larger multicenter studies are needed before broader conclusions or policy recommendations can be made.

## Data Availability

The datasets generated and/or analyzed during the current study are not publicly available due to patient confidentiality but are available from the corresponding author on reasonable request.
